# Immune-related adverse events in patients treated with immunotherapy for locally advanced or metastatic NSCLC in real-world settings: a systematic review and meta-analysis

**DOI:** 10.3389/fonc.2024.1415470

**Published:** 2024-07-09

**Authors:** Giulia Pasello, Alberto Pavan, Mattia De Nuzzo, Stefano Frega, Alessandra Ferro, Alessandro Dal Maso, Laura Bonanno, Valentina Guarneri, Fabio Girardi

**Affiliations:** ^1^ Department of Surgery, Oncology and Gastroenterology, University of Padova, Padua, Italy; ^2^ Oncologia 2, Istituto Oncologico Veneto (IOV) IRCCS, Padua, Italy; ^3^ Medical Oncology Department, Azienda ULSS 3 Serenissima, Dell’Angelo General Hospital, Mestre and SS Giovanni e Paolo General Hospital, Venice, Italy

**Keywords:** real world evidence, immunotherapy, immune-related adverse events, non-small cell lung cancer, meta-analysis

## Abstract

**Introduction:**

Randomized clinical trials (RCTs) represent the mainstay for the approval of new treatments. However, stringent inclusion criteria often cause them to depart from the daily clinical practice. Real-world (RW) evidence have a complementing role, filling the gap between the efficacy of a treatment and its effectiveness. Immune checkpoint inhibitors (ICIs) have changed the treatment scenario for non-small cell lung cancer (NSCLC); immune-related adverse events (irAEs) could become life-threatening events, when not timely managed. We performed a systematic review and meta-analysis on the RW impact of irAEs through the years.

**Methods:**

The systematic review focused on irAEs occurred in locally advanced or metastatic NSCLC patients, treated with ICIs in a RW setting. We queried two electronic databases (Embase and Medline) from 1996 to August 2022. We then conducted a meta-analysis dividing the results in two cohorts (2015-2018 and 2019-2021). We described the prevalence of patients with irAEs of any or severe grade (G). Estimates were expressed as proportions up to the second decimal point (effect size, ES). IrAEs of interest were those involving the skin, the liver, the endocrine system or the gastro-intestinal system.

**Results:**

Overall, 21 RW studies on 5,439 patients were included in the quantitative and qualitative synthesis. The prevalence of G≥3 irAEs was slightly lower in the 2015-2018 subgroup, while the prevalence of irAEs of any grade was similar for both periods. Overall, we observed a higher ES for gastrointestinal, hepatic and lung irAEs, while a lower ES was reported for skin or endocrine irAEs. Endocrine irAEs were reported in 10 out of 21 studies, with a slight increase in the most recent studies, while cutaneous toxicities were mostly reported in two studies lead within the first time-period. Pulmonary, gastrointestinal, and hepatic toxicities, showed a more heterogeneous distribution of ES over time.

**Discussion:**

Our findings showed that the frequency of irAEs remained stable across the two calendar periods examined in our meta-analysis. This finding suggests that RW data might not be able to identify a potential learning curve in detection and management of irAEs.

## Introduction

The clinical development and the regulatory approval of new drugs or new treatment strategies are currently based on the results of randomized clinical trials (RCTs), as they represent the main tool capable of generating the strongest form of evidence, for the assessment of the efficacy of an investigational therapeutic agent.

However, RCTs are not always able to answer all the research questions for healthcare decision makers. The reason is inborn in the nature of such trials, as they need to adopt strict inclusion and exclusion criteria, in order to minimize the risk of bias, causing them not to be fully representative of the patient population seen in the daily practice (1).

Hence, there is a constant need to quantify the entity of the gap between the efficacy of a treatment as per RCT and its real-world effectiveness, to study its transferability and sustainability in the daily clinical practice and also to better define the safety of a given treatment, especially in terms of practical management and long‐term adverse events.

Real-world evidence (RWE), defined as the analysis of real-world data, that is data relating to patients’ health and health care “routinely collected from a variety of sources”, could have a complementing role to data coming from traditional clinical trials.

In the field of lung cancer care, the advent of immune checkpoint inhibitors (ICIs) has dramatically changed the treatment scenario, improving patients’ outcome and establishing strategies including ICIs as the new standard of care (2–8).

We previously reviewed how the introduction of ICIs has caused an outbreak of RWE in lung cancer, with the number of real-world articles being three-times higher between 2015 (using July 2015 as a split point) and 2020, compared with the 2010-2015 period, before the breakthrough of ICIs in lung cancer care. Furthermore, we focused on special subsets of lung cancer patients, such as those affected by chronic viral diseases, diagnosed with brain metastases, or having a poor performance status. These patients have been traditionally excluded from pivotal RCTs and therefore data about the impact of ICIs on the outcome of this patient population are not available. RWE was able to produce valuable insights into the real treatment benefit for most of these subgroups (9).

With regards to safety profile, ICIs are well-tolerated drugs, characterized mostly by low-grade toxicities. However, immune-related adverse events (irAEs) linked with ICIs could also become life-threatening events, especially when not timely recognized and managed (10).

As we are approaching the landmark of the first decade of usage of ICIs in lung cancer, we set out to assess through a systematic review and a meta-analysis the impact of irAEs in the real world, with particular reference to the prevalence of different types of irAEs and the reporting trends through the years.

## Materials and methods

The systematic review focused on irAEs in patients treated with immunotherapy for locally advanced or metastatic non-small cell lung cancer (NSCLC) in a real-world setting. In order to identify eligible studies, we queried two electronic databases (Embase and Medline) from 1996 to 25^th^ of August 2022 (**Supplementary Table 1**). We used a pre-specified search strategy including terms for the disease domain, the study design domain and the treatment domain. We excluded all the articles published before 1996 because immunotherapy was not a standard of care in solid tumors in that period.

We also conducted a meta-analysis of real-world, observational studies on the prevalence of irAEs in patients affected by NSCLC and receiving immunotherapy with or without chemotherapy. For each relevant study, we extracted the raw number of events and the patient population size. These two parameters allowed the estimation of confidence intervals based on a binomial distribution. We expressed the prevalence of adverse events as a proportion up to the second decimal points. Here any given estimate was referred to as effect size (ES). We used the statistical package *metaprop*, as implemented in the software STATA18^®^. Pooled proportions were obtained for the prevalence of patients with irAEs of any grade, and for the prevalence of patients with grade 3 or higher (G≥3) adverse events among those developing immune-related toxicity. We considered irAEs all combined and by type. Events of interest were those occurring in the skin, the liver, the endocrine system or the gastro-intestinal system. Studies were also grouped by calendar year to investigate temporal trends in reporting of irAEs.

## Results

**Figure 1:**
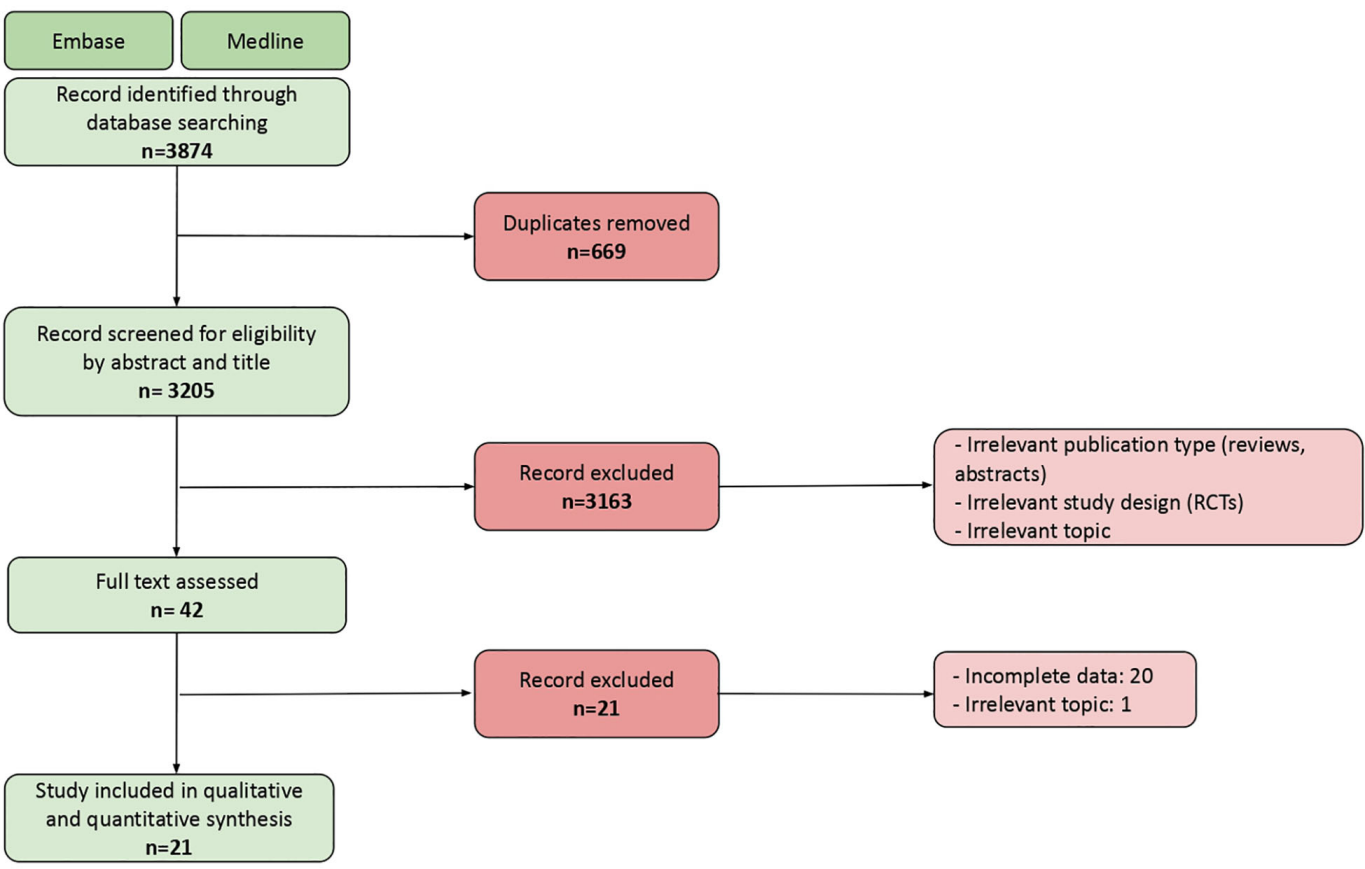
PRISMA flowchart showing the final number of studies included in the analysis, and main reasons for exlcusion.

The database search yielded 3205 records. We screened these records for eligibility by title and by abstract. We then assessed the full text of the remaining 42 studies. This process followed the Preferred Reporting Items for Systematic Reviews and Meta-Analyses (PRISMA) (11). The PRISMA flowchart is shown in **Figure 1**. Twenty-one real world studies reporting irAEs were therefore included in the quantitative and qualitative synthesis.

## Systematic review

We reviewed data from 21 selected real-world studies including a total of 5,439 patients with advanced-stage NSCLC treated with immunotherapy in consolidation, first-line, or subsequent therapy settings. The characteristics of the selected paper are summarized in **Table 1** (12–32).

**Table 1:** Characteristics of the studies included in the present meta-analysis.

First author	Study time	Sample size	Total irAE pts	G≥3 irAE pts	Total irAE	G≥3 irAE	characteristics of population			
							Median Age	PS≥2 (%)	Treatment line	Median FUP (months)
Grossi (12)	2015	371	109	21	168	24	68	5,93	≥2nd	7.5
Martin (13)	2016	109	NA	NA	229	12	65	15.6	≥2nd	8.83
Kobayashi (14)	2016	142	64	19	99	21	67	16.2	≥2nd	NA
Areses Manrique (15)	2017	188	146	9	155	9	58	10,00	1st and ≥2nd	NA
Bouhlel (16)	2017	69	31	4	53	5	63.3	20,30	≥2nd	13
Ricciuti (17)	2017	195	119	15	282	25	63	17,95	≥2nd	NA
Muchnik (18)	2017	75	28	6	37	6	37,3%≥ 80	49,33	1st and ≥2nd	NA
Ksienski (19)	2018	190	66	16	88	16	70	34.2	1st and ≥2nd	6.1
Yamaguchi (20)	2018	131	NA	NA	54	10	77	16,03	≥2nd	11.1
Matsubara (21)	2018	125	NA	NA	83	19	60	8,80	1st and ≥2nd	13,8
Ivanovic (22)	2018	66	52	8	97	20	64	6,00	1st and ≥2nd	up to 34,2
Chan (23)	2019	191	35	8	37	9	65	24,61	Consolidation, 1st and ≥2nd	NA
Altan (24)	2019	179	68	13	90	13	74,9	17,88	1st and ≥2nd	24,7
Ansel (25)	2019	82	60	6	60	6	67,37	2.4	1st	37
Isono (26)	2019	180	85	21	122	22	68.5	9,44	1st and ≥2nd	10
X. Chen (27)	2019	191	70	14	141	20	62	6,81	1st and ≥2nd	9,8
M. Chen (28)	2019	97	45	9	75	14	64	15,46	≥2nd	8,3
Tamayo- Bermejo (29)	2019	62	40	9	87	9	64	6,45	1st and ≥2nd	3
Ohe (30)	2020	2570	748	250	1171	316	69	18.1	≥2nd	NA
Alonso-García (31)	2020	158	102	23	243	23	64	15.2	≥2nd	8.32
Li (32)	2021	68	48	11	75	15	72	30.9	≥2nd	9,8

FUP, Follow up; G, Grade; irAE, Immune related Adverse Events; PS, Performance Status; PTS, patients; NA, not available.

As often occurs in real-world studies, the included populations encompassed a significant proportion (17%) of patients with an Eastern Cooperative Oncology Group Performance Status (ECOG PS) greater than or equal to 2 (PS≥2) and of advanced age (median age of 66 years old). This allowed the investigation of the efficacy and safety of immunotherapy in this particular patient subset, who is typically excluded or underrepresented in the pivotal clinical trials.

Several studies analyzed the efficacy of nivolumab in cohorts of pretreated patients (12–17). All the studies agreed that elderly patients with advanced NSCLC benefit from nivolumab, with tolerability similar to that in the overall population. According to Kobayashi et al. (14) an EGFR/ALK mutation-negative status and previous radiotherapy may be key clinical factors associated with a positive treatment response in this patient population.

All the authors agree that PS≥2 and disease burden are unfavorable prognostic factors in patients treated with immunotherapy. A negative correlation was found between patient PS and progression free survival (PFS), and some also believe that it significantly impact on overall survival (OS). The presence of brain metastases is recognized as an unfavorable prognostic factor by Manrique et al. (15). Similarly, Ksienski et al. (19) support the thesis that the number of metastatic sites can be a potential risk factor for predicting PFS in older patients.

A highly debated issue is whether the occurrence of irAEs during treatment is also associated with an improvement in OS. In this regard, the evidence from the studies selected for this review is conflicting. In particular, Ricciuti and Boulhel et al. (16, 17) agreed that patients who experienced irAEs had a more pronounced survival benefit compared to those with no irAE, further suggesting a causal association between irAEs and immunotherapy efficacy. Chen X. and Chen M. et al. (27, 28) highlighted that the occurrence of irAEs is correlated with a benefit in PFS, but not in OS. Additionally, Ksienski et al. (19) agreed that the occurrence of irAEs does not seem to increase OS.

Furthermore, three studies attempted to evaluate the efficacy and safety profile in “special” populations, which are also typically excluded from RCTs. Ansel et al. (25) analyzed the effect of Pembrolizumab in a small group of patients with autoimmune (AI) comorbidities. There was a trend towards better survival in patients with a previous AI comorbidity compared to those without. Nevertheless, irAEs were more common in patients with AI, but predominantly of low grade. In the study by Chan et al. (23), the use of ICIs was tested in patients with a history of tuberculosis (pTB) and/or chronic inactive hepatitis B virus (HBV). Immune checkpoint inhibition proved to be a safe and effective option that can be considered also in patients with advanced NSCLC who have a history of pTB and HBV. There was no increased all-grade adverse events in this group of patients and no significant flare of underlying infections. Isono et al (26). described a particular cohort of patient with pre-existing respiratory disease, including idiopathic interstitial pneumonias treated with ICIs. In this case, pneumonitis was the most frequent adverse event, but we have also to consider that a part (18.3%) of these patients had also previously received thoracic radiotherapy.

In Alonso-Garcia et al. (31), the safety and efficacy outcomes of atezolizumab or nivolumab in lines of therapy subsequent to the first were considered. The safety results suggested a less favorable profile for nivolumab compared to atezolizumab. Regarding immune-related adverse effects, the most common were skin disorders (including pruritus and rash) and pneumonitis. Tamayo-Bermejo et al. (29) analyzed the efficacy and safety of pembrolizumab in a more selected population (patients less than 65 years old and a PS 0-1): no grade 4 adverse reaction was reported.

Li et al. (32) analyzed the effectiveness and safety of PD-1 inhibitor monotherapy in a cohort of elderly patients with advanced NSCLC: 70.6% patients had irAEs of any grade and for 16.2% of the patients adverse reactions were G≥3. One patient died from the PD-1 inhibitor-related pneumonitis. Abnormal liver function was the most frequent grade 3 irAE, followed by diarrhea (2.9%), nausea and vomiting (2.9%), rash (1.5%), and pneumonitis (1.5%). Despite the toxic death, the overall safety profile was deemed as favorable by the authors. Also Altan et al. (24) identified pneumonitis as the cause of a therapy-related death for one patient. However, in this study only 38% of the patients experienced irAEs with ICI therapy, and 7.2% of the patients that had G≥3 irAEs. Pneumonitis was the most common severe irAE and the main reason leading to treatment discontinuation.

## Meta-analysis

For the purpose of investigating temporal trends in irAE reporting, for every study, we used the latest calendar year for inclusion of the patients in the study, referred to as “study time” in **Table 1**. The articles were divided into two different groups: one including the studies with a deadline for enrolment between 2015 and 2018, the other with the deadline between 2019 and 2021.

**Figure 2:**
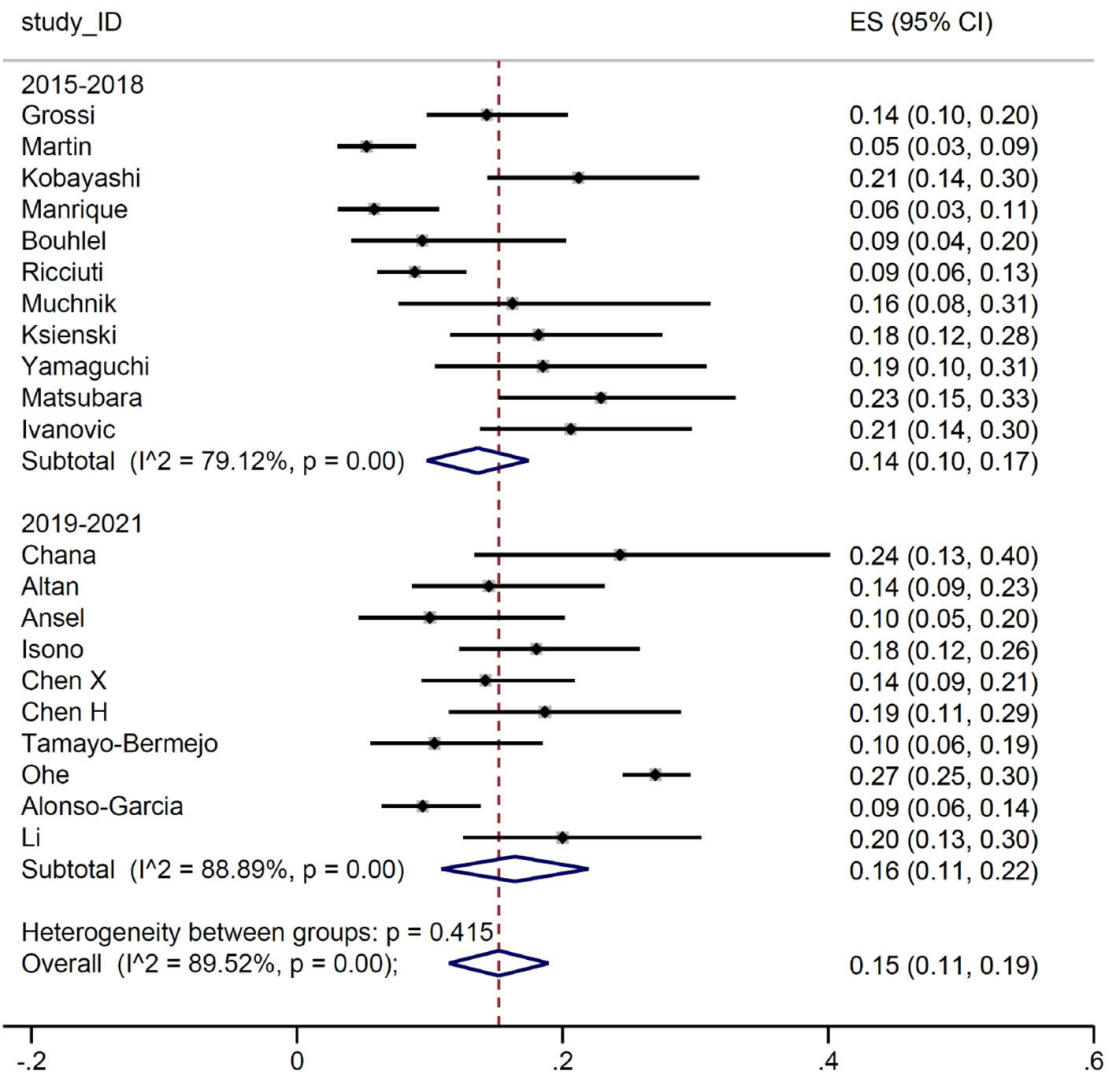
Grade ≥ 3 adverse events by study time.

The estimated ES for G≥3 adverse events was slightly lower in the 2015-2018 subgroup than in the 2019-2021, 0.14 *versus* (*vs*) 0.16. However, given the heterogeneity between both the studies and the subgroups, confidence intervals (CI) were widely overlapping (95% CI 0.10-0.17 *vs* 95% CI 0.11-0.22) (**Figure 2**).

**Figure 3:**
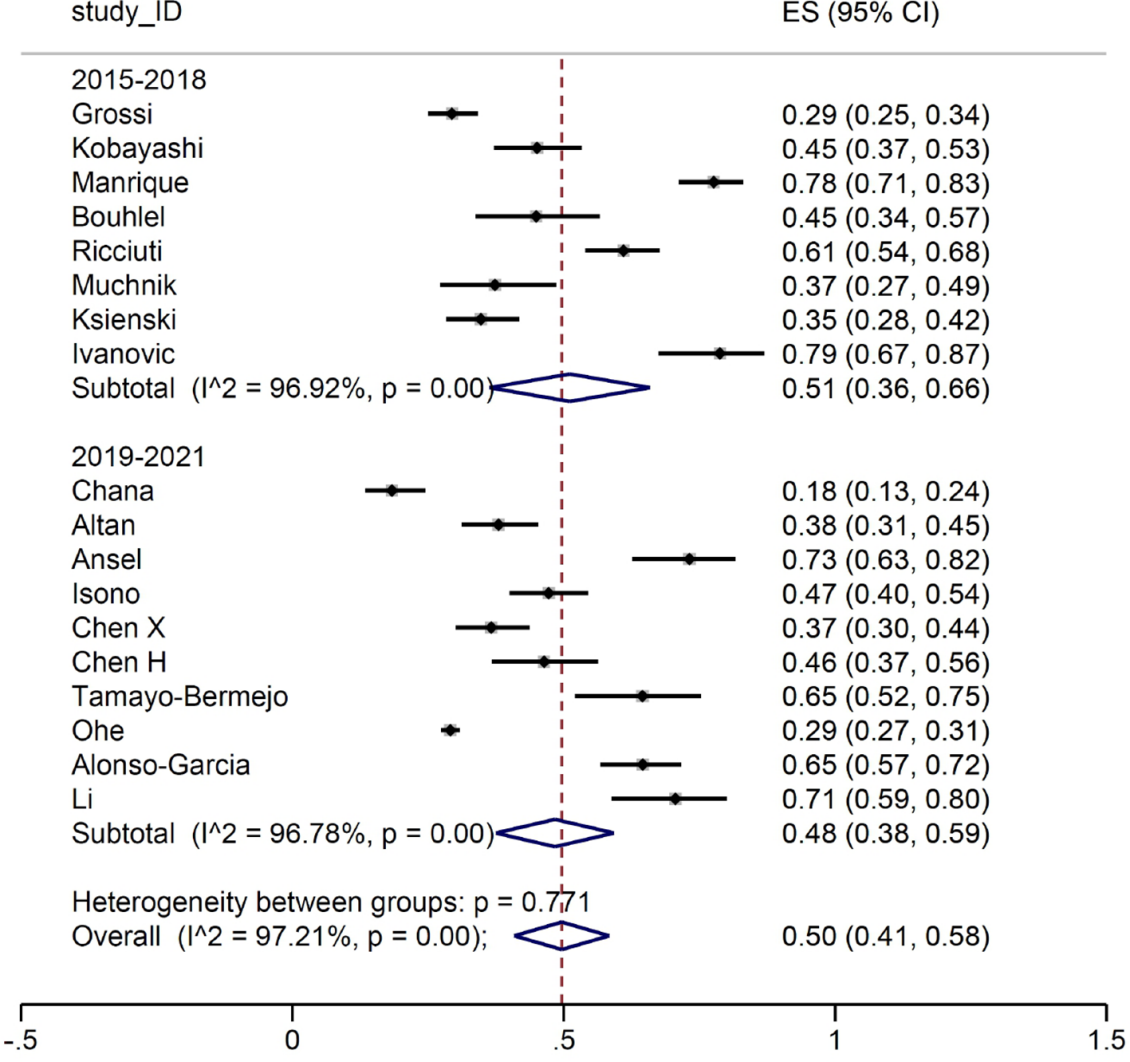
Patients with immune-related adverse events of any grade.

When we focused on the patients developing irAEs of any grade, no difference was observed between the two study periods (**Figure 3**).

**Figure 4:**
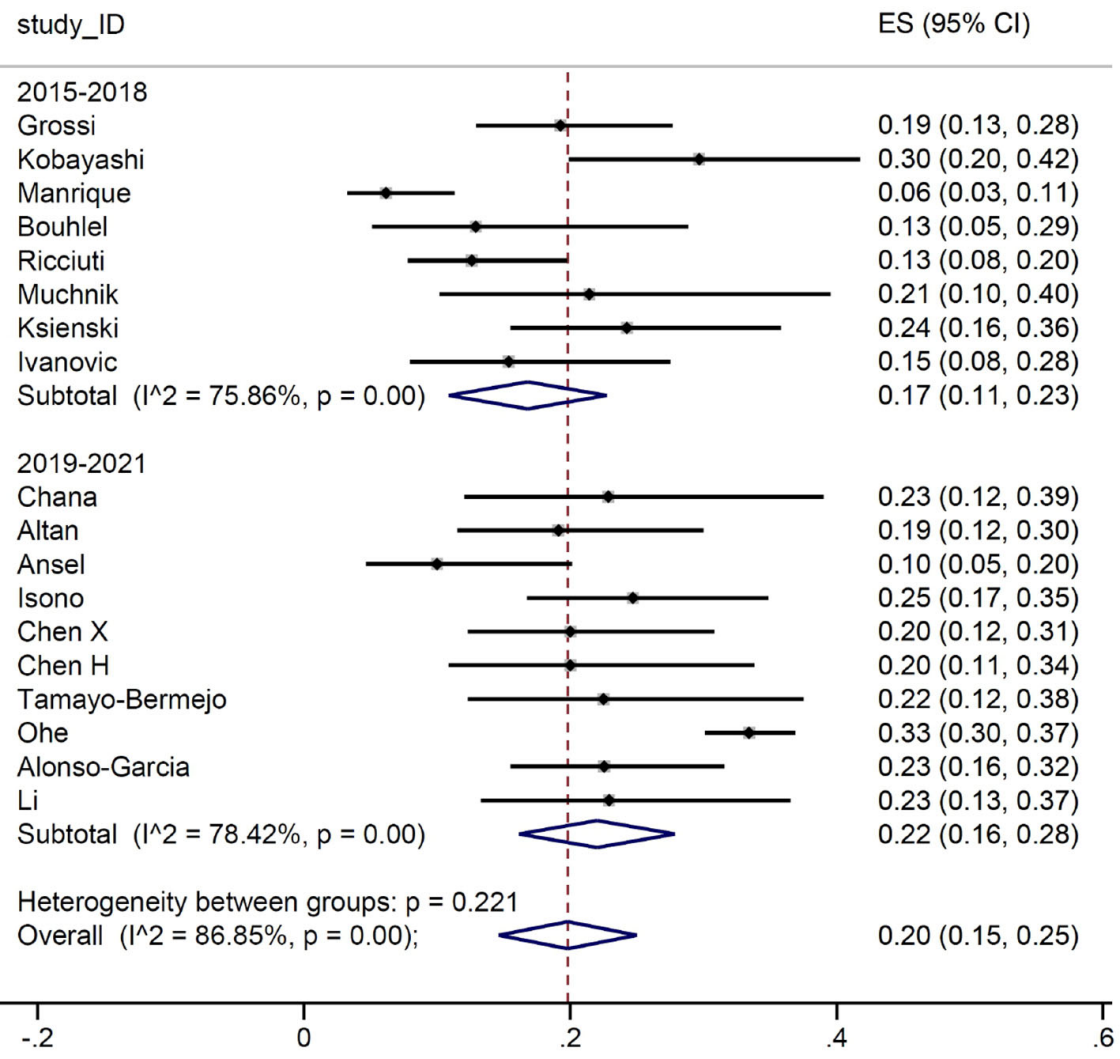
Grade ≥ 3 immune-related adverse events by study time. .

The difference between the two groups was more pronounced when we look at the number of patients who have a G≥3 irAEs, as we can see in **Figure 4**.

In addition, we examined the trends of different types of G3 or more irAEs by anatomical site. We considered immune-related pneumonitis, gastrointestinal toxicities (diarrhea, colitis, nausea, and vomiting), skin toxicity (rash, pruritus), hepatic toxicity (elevation of transaminases and bilirubin), endocrine toxicities (thyroiditis, hypothyroidism, hyperthyroidism, endocrinopathy, hyperglycemia, hypophysitis). For each toxicity subgroup, studies were excluded if the specific adverse event was not reported.

We observed a higher ES for gastrointestinal (ES 0.22, 95% CI 0.13-0.31), hepatic (ES 0.31, 95% CI 0.20-0.41) and lung irAEs (ES 0.35, 95% CI 0.26-0.43, while a lower ES was reported for skin (ES 0.06, 95% CI 0.03-0.08) and endocrine irAEs (ES 0.09, 95% CI 0.05-0.12) (**Supplementary Figures 1–5**).

Severe endocrine irAEs were reported in only 10 out of the total 21 studies, with a slight increasing trend in the most recent calendar period. Severe skin toxicities were slightly more common during 2015-2018 than in 2019-2021, but this finding was only based on two studies. Lung, gastrointestinal, and hepatic toxicities, conversely, showed a more heterogeneous distribution in terms of effect size over time.

## Discussion

RWE has become a source for a new kind of scientific evidence complementary to the one derived from interventional clinical trials. If the latter is capable of producing perfect data from highly selected and therefore almost “imperfect” patients, the former is often described as offering imperfect data from perfect patients, namely those usually seen in the daily practice.

In this scenario, RW data could be very informative and helpful, especially when new treatment strategies subvert the standard of care and clinical practitioners must become accustomed to a completely different class of drug, both in terms of mechanism of action and of safety profile.

With this work, we decided to focus on real-world studies, including advanced NSCLC patients who received immunotherapy and presenting data about immune-related toxicities. Following a strict screening process, we selected 21 real world studies, dating back to 2015, the year of the outbreak of ICIs for NSCLC and the dawn of the RW research in lung cancer care.

The findings of the 21 clinical studies showed that immune checkpoint inhibition represents an effective and well-tolerated therapeutic option for patients with advanced NSCLC. Most of the studies agreed in defining a larger benefit in terms of outcome for patients with performance status (PS) 0-1 compared to those with PS of 2 or more. While for some studies this advantage was only found in progression-free survival (PFS) (24, 27, 28), other authors (13, 19, 31) also highlighted a remarkable advantage also in overall survival (OS). Similarly, other real-world studies defined the extent of disease as a key prognostic factor for patients treated with immunotherapy. This aspect is particularly addressed by Ksienski et al. (19), who asserts that disease burden expressed as the number of metastatic lesions can be a potential negative prognostic factor in predicting the PFS of older patients. According to Manrique et al. (15), CNS involvement is a prognostically negative factor, as are bone and liver involvement in the work by Grossi et al. (12). These data are consistent with other real-life studies recently published on immunotherapy in patients with advanced NSCLC (33, 34).

The incidence and severity of adverse events reported across the studies including diarrhea, rash, pneumonitis, endocrine disorders and elevated liver enzymes may vary between studies and patient populations, but their spectrum and timing remain consistent among RW studies and with randomized trials for NSCLC. Whether the occurrence of these irAEs is a prognostically favorable factor in patients treated with ICI is still controversial. Martin, Ricciuti, Isono, and Bouhlel et al. (13, 16, 17, 26) agree that there is an advantage in terms of outcome in patients who develop irAEs during treatment.

We then conducted a meta-analysis on the prevalence of irAEs, dividing the study by calendar period. First, we considered the occurrence of G≥3 irAE: no substantial differences were detected between the two groups, with a proportion of events between 14% and 16%. Subsequently, we focused on the number of patients developing irAEs. Again, the prevalence of reported any-grade immune-related toxicities was almost identical for the two groups (51% and 50%, respectively). Such result is in line with the evidence from the main RCTs, where the prevalence of any-grade irAEs ranged between 62% and 76%. Similarly, when we considered patients with G≥3 irAEs, the estimates (17% and 20%, respectively) were comparable with those reported in RCTs, ranging between 10.8% and 33%. It is of interest that the frequency of irAEs remained stable across the two periods examined in our meta-analysis. Indeed, although our initial hypothesis was that there might be a lower incidence of severe adverse events in the second triennium due to improved early detection and management, this was not the case. This finding allows us to conclude that real-world data collection from observational reports does not address the question of a potential learning curve in detection and management of irAEs.

On the other hand, it is also true that increased awareness could lead to more frequent classification of adverse events as severe, possibly due to greater diagnostic intensity and reporting.

In order to achieve more reliable information on adverse events occurring in the daily practice, the prospective collection of data from real-world studies with those from pharmacovigilance registries, should be aimed.

Moreover, we evaluated the prevalence of different subtypes of irAEs, regardless of the calendar period. We focused on five main categories: immune-related hepatic, gastro-enteric, lung and skin AEs and immune-related endocrinopathies.

The prevalence of hepatic IrAEs rate was 31% (95% CI 20-41%). This value is slightly higher than the ones reported in mono-immunotherapy RCTs (prevalence ranging between 1.0% and 20.3%) (2–8) and remarkably higher than the prevalence reported in a recent meta-analysis of all phase III clinical trials assessing ICIs for lung cancer (6.2%) (35). Similarly, gastro-enteric irAEs in our study accounted for 22% (95% CI 13%-31%), whereas their impact in mono-ICI trials ranged between 6 and 20.1% and in the all-comers meta-analysis accounted for 15.1% of all irAEs. A possible explanation might reside in the ECOG PS of the patients included in our study, especially those with a PS≥2, who represented almost 17% of all the study population. Such characteristic has been reported to be linked with higher risk of irAEs development (36, 37).

The prevalence of skin-related AEs and immune-related endocrinopathies was 6% (95% CI 3-8%) and 9% (95% CI 5-12%) respectively, both below the incidence-range described in literature. The reason might lie in the fact that 7 out of 21 (33.3%) and 10 out of 21 (47.6%) articles did not report data about skin-related and endocrine irAEs, respectively.

It is undeniable that one of the main current limitations of RWE lies within its quality. A recently presented systematic revision regarding RWE on oncology targeted therapies in the 2022-2023 time-frame showed that, among 1251 studies, the majority (40%) were single-center experiences and that only 3% were published in high impact journals (38). With the purpose of improving the quality of RWE, several guidelines have been developed. One of the most complete guidelines about RWE is ESMO Guidance for Reporting Oncology Real-World evidence (GROW) (39). The publication of this article is likely to have several consequences for systematic reviews dealing with real-world evidence. Firstly, it can enhance the quality of reviews by providing clear standards for evaluating RWE studies in oncology and ensuring a more rigorous and transparent approach to evidence synthesis. Secondly, it may lead to standardized reporting practices, facilitating comparability and synthesis of data across studies. Lastly, it may reduce variability in reporting, promoting consistency in how RWE studies are assessed and interpreted within systematic reviews.

In July 2023, EMA published a report on the “Real-world evidence framework to support EU regulatory decision-making”, evaluating the experience gained with regulator-led studies from September 2021 to February 2023. In 63% of cases, study results were supportive and consequently considered for the assessment in the decision-making process. Furthermore, the report highlighted both the need of collecting a wider range of date, coming from complementary data sources, and the need to accelerate the generation of RWE, developing a common precomputed data model in order to reduce the time needed for the completion of more advanced (and thus time-consuming) data analyses.

The added value of the present work is the attempt of summarize the huge amount of real-world data about the safety of ICIs and to meta-analyze them in order to offer to clinicians an overview of temporal trend in detection and reporting. Our data integrate available data from RCTs within an innovative model of drug clinical development and underline the importance of well-designed RW studies according to reliable methodologies, where clinical data are matched with health flows and registries (40).

## Data availability statement

The original contributions presented in the study are included in the article/**Supplementary Material**. Further inquiries can be directed to the corresponding author.

## Author contributions

GP: Writing – review & editing, Writing – original draft, Supervision, Methodology, Funding acquisition, Data curation, Conceptualization. AP: Writing – review & editing, Writing – original draft, Validation, Investigation, Data curation. MD: Writing – original draft, Project administration, Investigation, Formal analysis, Data curation. SF: Writing – review & editing, Visualization, Investigation, Data curation. AF: Writing – review & editing, Software, Project administration, Methodology, Formal analysis, Data curation. AD: Writing – review & editing, Visualization, Validation, Resources, Investigation, Data curation. LB: Writing – review & editing, Visualization, Validation, Supervision, Resources. VG: Writing – review & editing, Visualization, Supervision, Methodology, Funding acquisition. FG: Writing – review & editing, Writing – original draft, Validation, Supervision, Software, Methodology, Investigation, Formal analysis.
