# Supplementary material for: Immune-related adverse events in patients treated with immunotherapy for locally advanced or metastatic NSCLC in real-world settings: a systematic review and meta-analysis
Source: Front Oncol. 2024 Jul 9;14:1415470. doi: 10.3389/fonc.2024.1415470 (PMC11263096; doi:10.3389/fonc.2024.1415470)

**Supplementary table 1: Search contents and results of eligible studies for the review from the Embase and Medline databases.**

| # | Search contents | n° of results |
| --- | --- | --- |
| 1 | lung cancer.mp. or exp lung cancer/ | 628,169 |
| 2 | lung carcinoma.mp. or exp lung carcinoma/ | 192,636 |
| 3 | lung neoplasms.mp. or exp lung tumor/ | 567,587 |
| 4 | (lung cancer or lung cancer or (lung carcinoma or lung carcinoma) or (lung neoplasms or lung tumor)).af. | 601,835 |
| 5 | exp immunotherapy/ or immunotherapy.mp. or exp cancer immunotherapy/ | 529,697 |
| 6 | clinical trial.mp. or exp clinical trial/ | 2,746,974 |
| 7 | randomized controlled trial.mp. or exp randomized controlled trial/ | 1,428,571 |
| 8 | controlled clinical trial.mp. or exp controlled clinical trial/ | 1,391,763 |
| 9 | (clinical trial or clinical trial or (randomized controlled trial or randomized controlled trial) or (controlled clinical trial or controlled clinical trial)).af. | 2,805,668 |
| 10 | exp toxicity/ or toxicit*.mp. | 1,757,894 |
| 11 | adverse events.mp. or exp adverse event/ | 956,928 |
| 12 | (toxicity or toxicit* or (adverse events or adverse event)).af. | 2,102,121 |
| 13 | ((((lung cancer or lung cancer or (lung carcinoma or lung carcinoma) or (lung neoplasms or lung tumor)) and (immunotherapy or immunotherapy or cancer immunotherapy)) not (clinical trial or clinical trial or (randomized controlled trial or randomized controlled trial) or (controlled clinical trial or controlled clinical trial))) and (toxicity or toxicit* or (adverse events or adverse event))).af. | 4,332 |
| 14 | animal*.mp. [mp=ti, ab, hw, tn, ot, dm, mf, dv, kf, fx, dq, bt, nm, ox, px, rx, an, ui, sy] | 8,966,683 |
| 15 | (((((lung cancer or lung cancer or (lung carcinoma or lung carcinoma) or (lung neoplasms or lung tumor)) and (immunotherapy or immunotherapy or cancer immunotherapy)) not (clinical trial or clinical trial or (randomized controlled trial or randomized controlled trial) or (controlled clinical trial or controlled clinical trial))) and (toxicity or toxicit* or (adverse events or adverse event))) not animal*).af. | 3,874 |
| 16 | remove duplicates from 15 | 3,205 |

**Supplementary figure 1. G≥3 gastrointestinal immune-related adverse events**


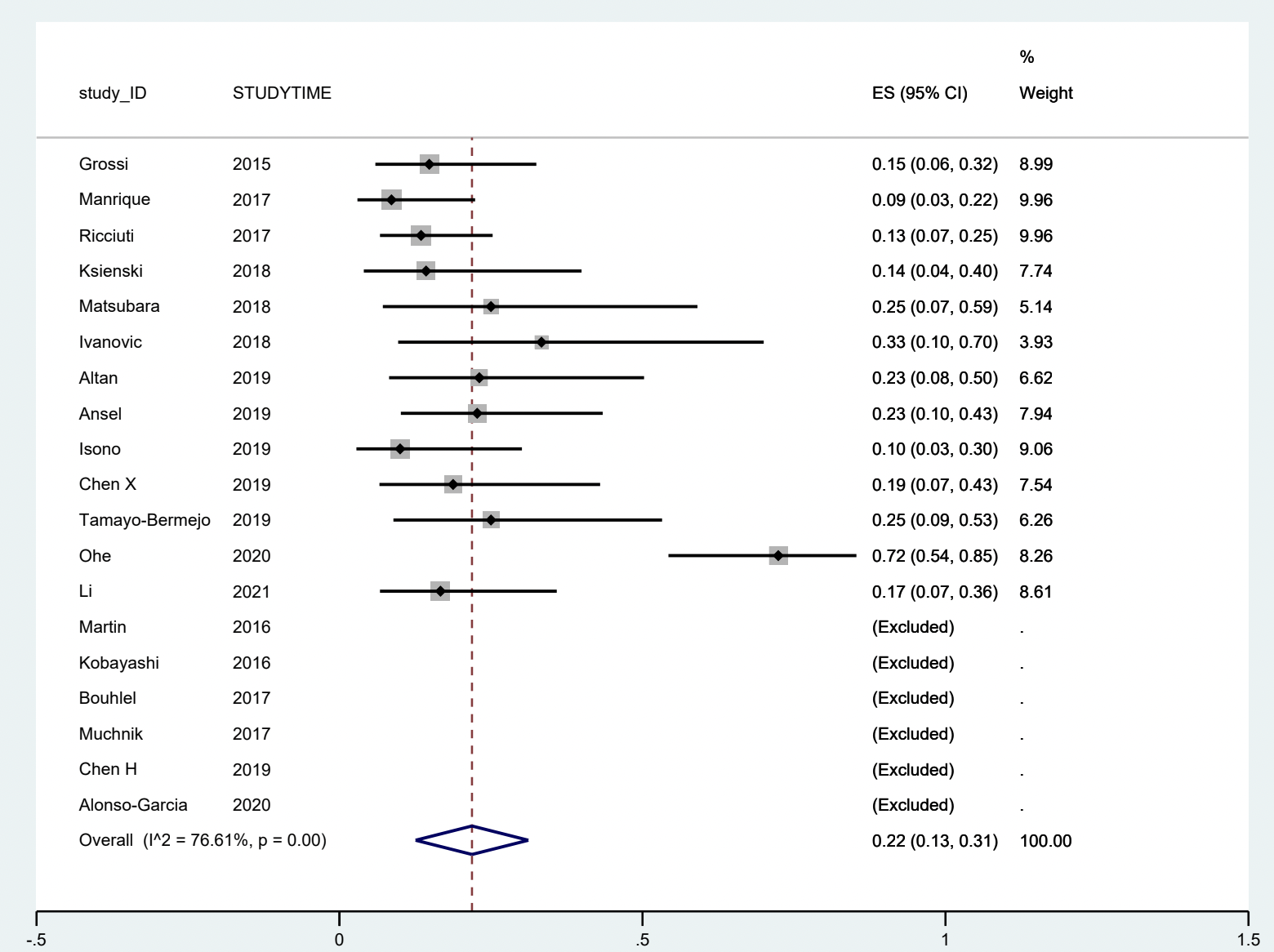


**Supplementary figure 2. G≥3 hepatic immune-related adverse events**

**
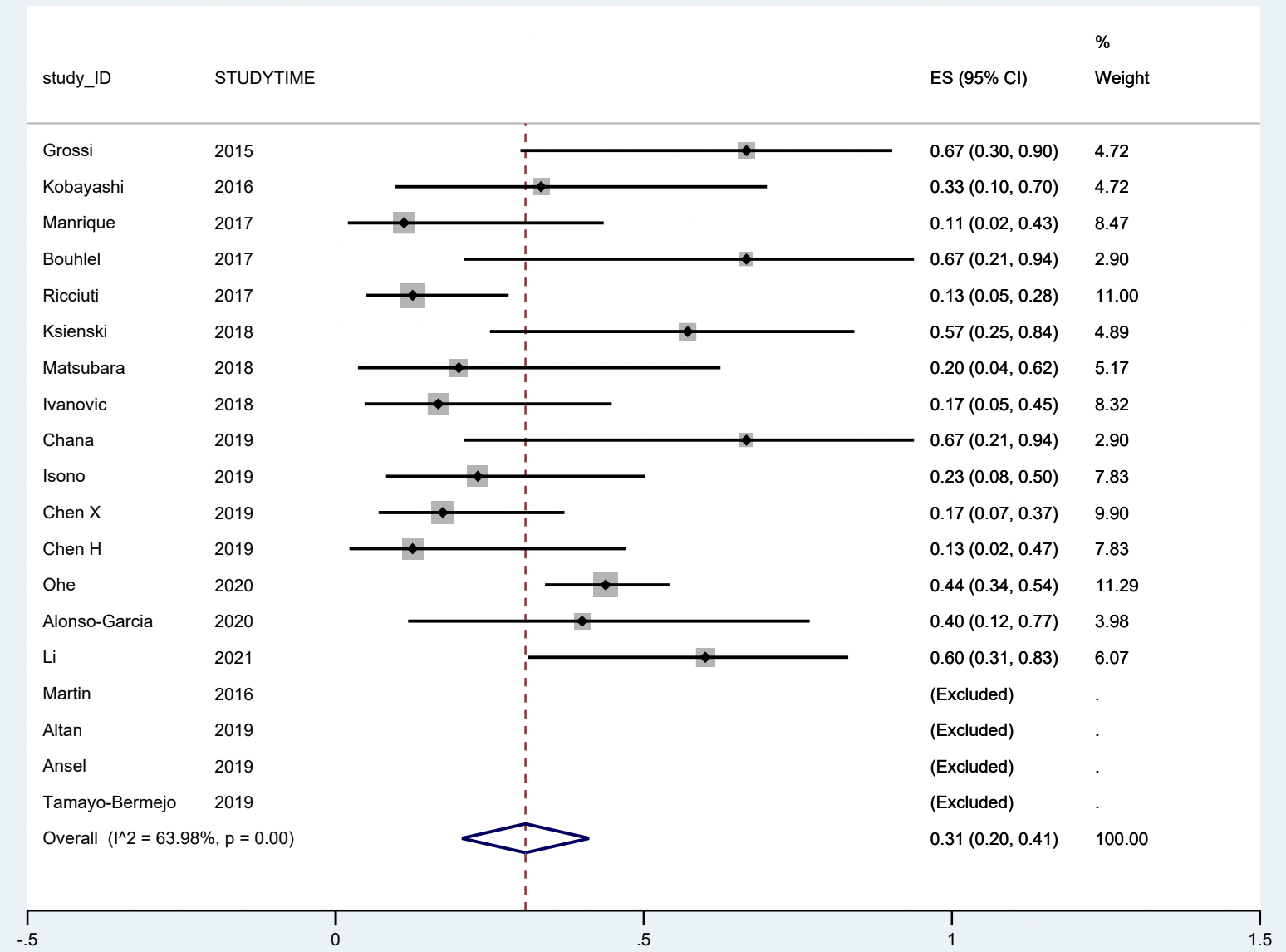
**

**Supplementary figure 3. G≥3 lung immune-related adverse events**


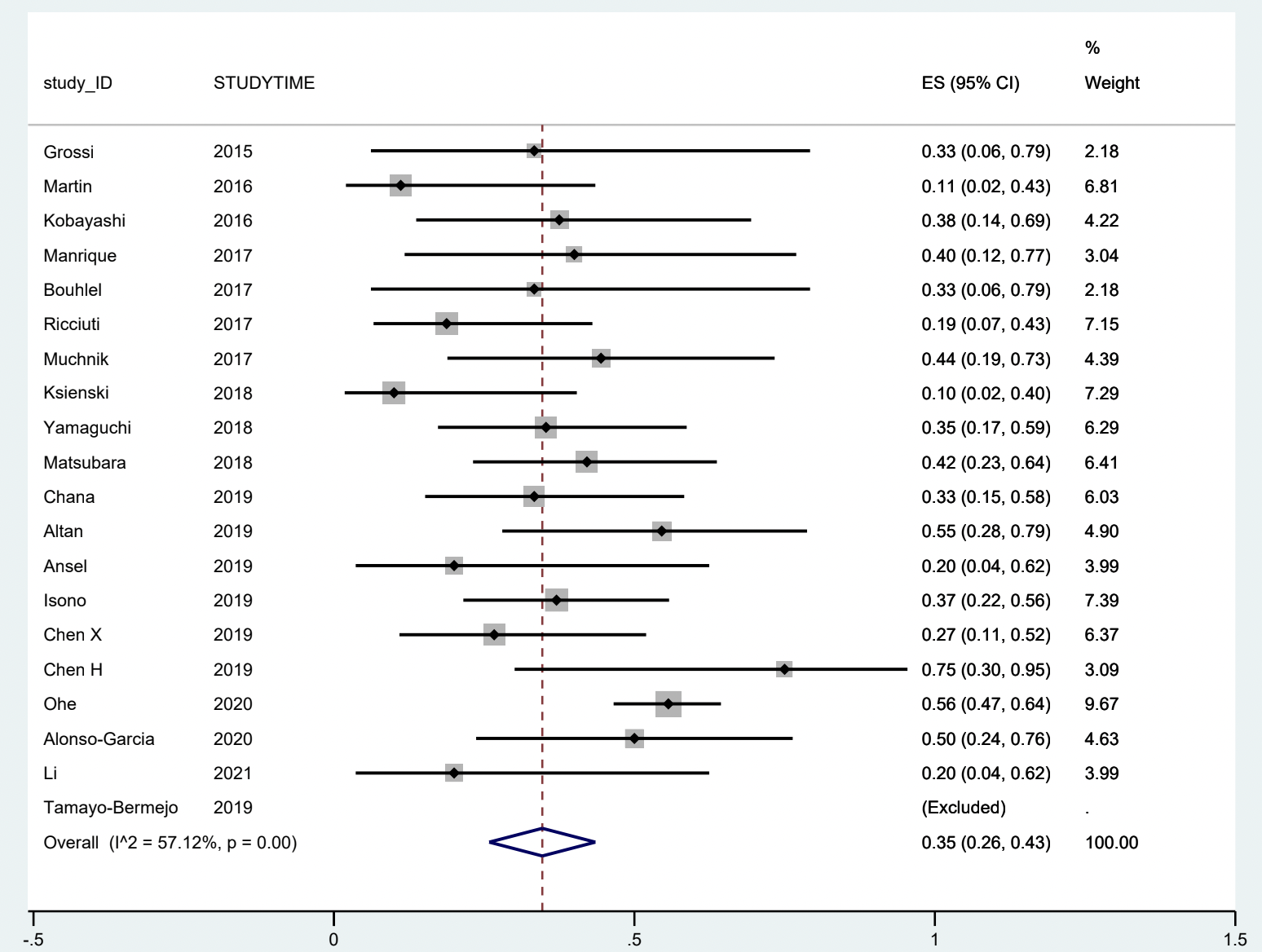


**Supplementary figure 4. G≥3 skin immune-related adverse events**

**
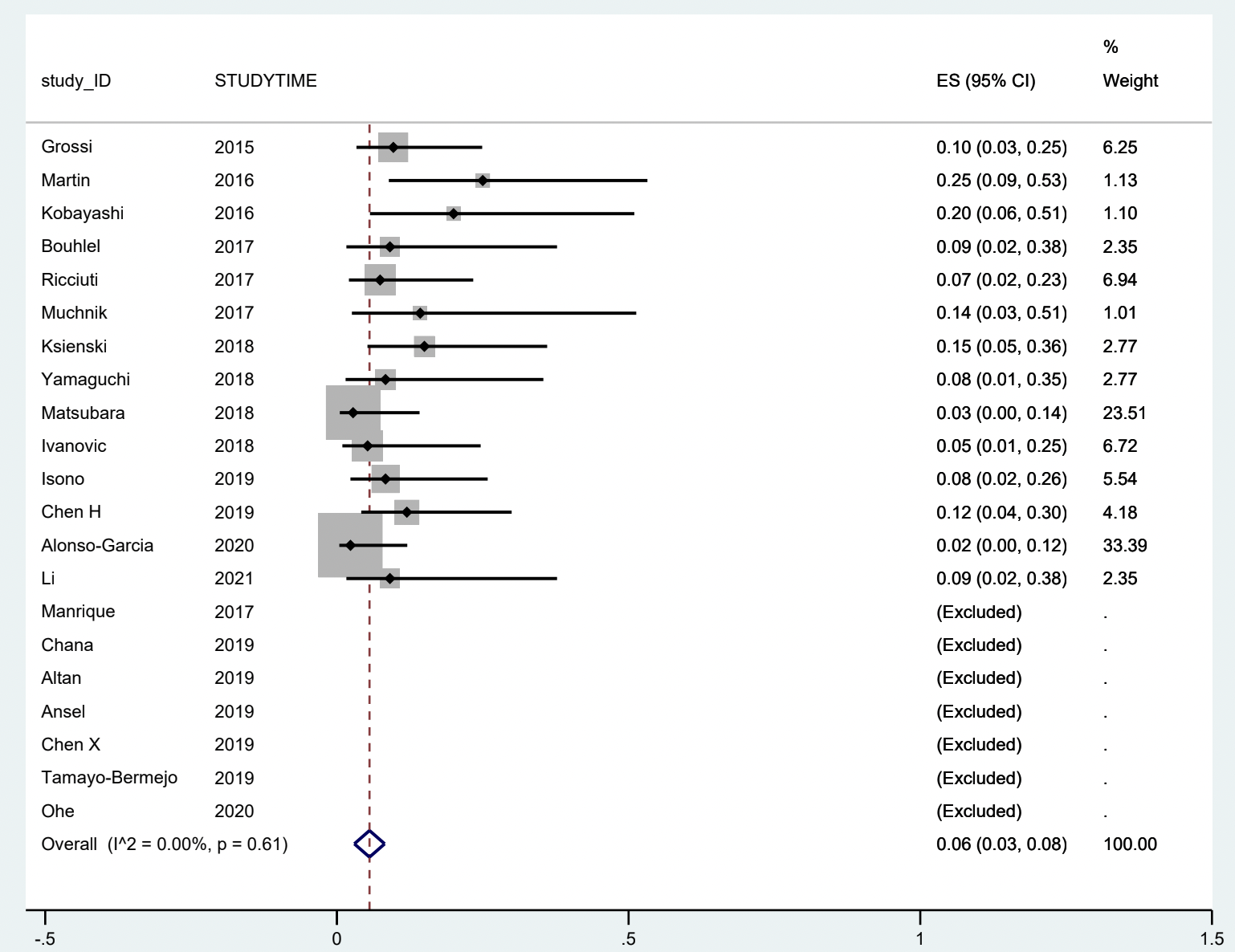
**

**Supplementary figure 5. G≥3 endocrine immune-related adverse events**


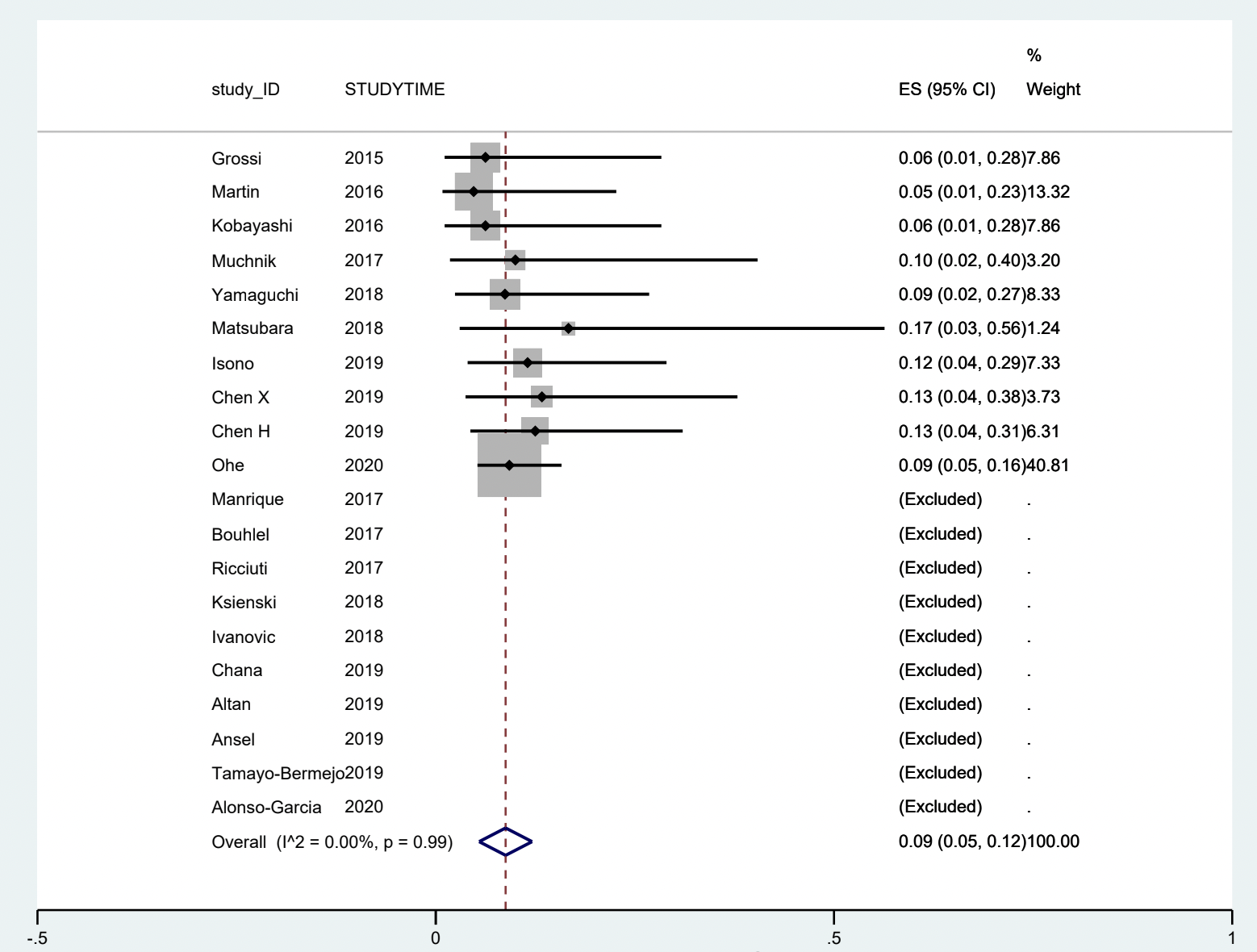

Supplement: Supplementary file 1 [file DataSheet_1.docx]
